# Functional Connectivity Alterations along the Longitudinal Axis of the Hippocampus in Aging and Alzheimer's Disease

**DOI:** 10.1002/alz70856_101978

**Published:** 2025-12-25

**Authors:** Esra Kara, Julian Dronse, Gereon R. Fink, Oezguer A. Onur

**Affiliations:** ^1^ University of Cologne, Faculty of Medicine and University Hospital Cologne, Cologne, Germany; ^2^ Cognitive Neuroscience, Institute of Neuroscience and Medicine (INM‐3), Research Center Jülich, Jülich, Germany

## Abstract

**Background:**

The hippocampus, a key region of the limbic system, plays a pivotal role in cognitive decline in Alzheimer's disease (AD). Previous neuroimaging studies revealed that the hippocampus should not be seen as a homogeneous structure but is instead distinctively organized along its longitudinal axis. However, how the organization of the hippocampus along its anterior‐posterior axis is involved in functional connectivity alterations in AD remains elusive.

**Method:**

We studied 26 healthy young subjects (HY, mean age 22.9 ± 1.38), 27 healthy seniors (HS, mean age 68.8 ± 6.8 years) and 33 patients with symptomatic Alzheimer's disease (AD, mean age 62.7 ± 6.5 years). Anterior and posterior hippocampal masks were derived from manual tracing and segmentation according to the EADC‐ADNI harmonized protocol. The individual hippocampal subregion masks were used as subject‐specific seeds for a seed‐based functional connectivity analysis of resting‐state functional magnetic resonance imaging (fMRI) data. Between‐group differences in functional connectivity regarding components of the default‐mode network were assessed using a contrast‐based approach.

**Result:**

The comparison between healthy young and healthy senior subjects revealed no substantial differences in functional connectivity to the precuneus, both in analyses using the entire hippocampus masks as seeds and in analyses contrasting the anterior with the posterior part. Applying a seed comprising all parts of the hippocampus showed no significant differences in functional connectivity to the precuneus when comparing healthy seniors and patients with AD (corrected for age). A distinct effect between groups was observed when using the anterior and posterior hippocampal subregions as separate seeds.

**Conclusion:**

We observed a loss of differentiation in connectivity from the hippocampus to the precuneus, an integral node of the default mode network, in AD. Changes in hippocampal functional connectivity may serve as a valuable, early neuroimaging biomarker in AD, particularly distinguishing physiological aging from pathological processes. The functional distinction of the hippocampus along its anterior‐posterior axis should be embedded in future analyses of these functional connectivity alterations.

